# Supplementary material for: Multi-OMICs landscape of SARS-CoV-2-induced host responses in human lung epithelial cells
Source: iScience. 2022 Dec 28;26(1):105895. doi: 10.1016/j.isci.2022.105895 (PMC9794516; doi:10.1016/j.isci.2022.105895)
Supplement: Document S1. Figures S1–S5 and Tables S1 and S2 [file mmc1.pdf]

iScience, Volume *26*

Supplemental information

Multi-OMICs landscape

of SARS-CoV-2-induced host responses

in human lung epithelial cells

Sneha M. Pinto, Yashwanth Subbannayya, Hera Kim, Lars Hagen, Maria W. Górna, Anni I. Nieminen, Magnar Bjørås, Terje Espevik, Denis Kainov, and Richard K. Kandasamy

Supplementary Data

Supplementary Tables

Table S1 related to Figure 1 and Figure 2.
List of differentially regulated transcripts identified in Calu-3 cells infected with SARS-CoV-2/ Trondheim-S15/2020 strain at time intervals 3-, 6-, 12-, 24-, and 48-hours post-infection (hpi).

Table S2 related to Figure 1 and Figure 2. List of differentially expressed proteins identified in Calu-3 cells infected with SARS-CoV-2/ Trondheim-S15/2020 strain at time intervals 3-, 6-, 12-, 24-, and 48-hours post-infection (hpi).

Table S3 related to Figure 1 and Figure 3. List of differentially phosphorylated proteins and phosphosites identified in Calu-3 cells infected with SARS-CoV-2/ Trondheim-S15/2020 strain at time intervals 3-, 6-, 12-, 24-, and 48-hours post-infection (hpi).

Table S4 related to Figure 1 and Figure 3. List of differentially acetylated proteins and acetylation sites identified in Calu-3 cells infected with SARS-CoV-2/ Trondheim-S15/2020 strain at time intervals 3-, 6-, 12-, 24-, and 48-hours post-infection (hpi).

Table S5 related to Figure 3. Pathways enriched in the 8 phosphoproteome clusters

Table S6 related to Figure 1 and Figure 4. Exometabolomics data from Calu-3 cells infected with SARS-CoV-2 at time intervals 3-, 6-, 12-, 24-, and 48-hours post-infection (hpi).

Table S7 related to Figure 5. Viral omics profile from Calu-3 cells infected with SARS-CoV-2 at time intervals 3-, 6-, 12-, 24-, and 48-hours post-infection (hpi).

Supplementary Figures

Supplementary Figure S1 related to Figure 1. Correlation graphs comparing **A.** transcriptome and **B.** proteome results of uninfected Calu-3 cells across our and other studies. Correlation graphs depicting replicate samples and various time points for SARS-CoV-2/ Trondheim-S15/2020 strain infected Calu-3 cell **C.** transcriptome **D.** total proteome, **E.** phosphoproteome, and **F.** acetylome data.

Supplementary Figure S2 related to Figure 2. A. Biological Processes (GO-BP) enriched in the Calu-3 transcriptome and proteome after SARS-CoV-2 infection. **B.** An Upset graph showing overlap of differentially expressed proteins in lung cell lines in response to SARS-CoV-2 infection between the current study and various published datasets, including Thorn *et al.*, Hekman *et al.*, Grossegesse *et al.*, and Stukalov *et al.* **C.** Heatmap depicting transcript levels of cytokines and chemokines in Calu-3 cells at time intervals 3, 6-, 12-, 24-, and 48-hours post-infection (hpi). **D.** A scan of the cytokine array showing changes in levels of cytokines in Calu-3 cells infected with SARS-CoV-2/ Trondheim-S15/2020 strain at time intervals 3, 6-, 12-, 24-, and 48-hours post-infection (hpi).

Supplementary Figure S3 related to Figure 3. A. An Upset graph showing overlap of hyperphosphorylated proteins in lung cell lines in response to SARS-CoV-2 infection between the current study and various published datasets, including Thorn *et al.*, Hekman *et al.*, and Stukalov *et al.* **B.** Heatmap showing enriched upstream kinases from the phosphoproteomics profile of Calu-3 cells after SARS-CoV-2 infection **C.** Statistics of differentially phosphorylated transcription factors in response to SARS-CoV-2 infection **D.** Heatmap showing changes in the levels of transcription factors after SARS-CoV-2 infection. **E.** Graph showing changes in Lysine (K) acetylation sites, Serine (S) phosphorylation sites and protein abundance of Vimentin (VIM). **F.** Graph showing changes in Lysine (K) acetylation and ubiquitination sites of leucine rich repeat containing 59 (LRRC59)

Supplementary Figure S4 related to Figure 4. Comparison of intracellular [94,95] and extracellular levels of **A, B** glycolysis metabolites, **C, D.** TCA cycle metabolites and **E, F.** amino acids

Supplementary Figure S5 related to Figure 5. Phosphorylation sites unique to this study are presented as magenta sticks, whereas common sites are in cyan. Experimental structural models of **A.** ORF7a (PDB ID 6w37), **B.** ORF8 (PDB ID 7jx6), **C.** ORF9b (PDB ID 7kdt) and Robetta models of **D.** N protein **E.** ORF3a, **F.** ORF6, **G.** Rep1a, **H.** Nsp3, and **I.** Protein M are shown in rainbow cartoon representation (N-to-C blue to red). Missing regions of Protein N and Rep1a Nsp1 were modelled with Robetta and presented as a rainbow cartoon, and were hidden where experimental structures of Protein N (PDB ID 6vyo for NTD, in olive and PDB ID 6wzo for CTD, in grey) and Rep1a Nsp1 (PDB ID 7k7p for NTD, in teal and PDB ID 7jqb for CTD, in purple) were overlapping with the models. The second protomer of ORF3a (PDB ID 7kjr in grey) and Tom70, the interaction partner of ORF9b (PDB ID 7kdt, in tan) are shown in uniform colors. Selected hydrogen bonds are shown as cyan dotted lines. The figure was prepared with ChimeraX (Goddard et al doi: 10.1002/pro.3235)

Supplementary Figure S6 related to Figure 6. Graphs showing the average trend of differentials from transcriptomics, proteomics, and phosphoproteomics datasets with respect to various pathways and processes, including **A.** Alternative splicing by spliceosome **B.** Regulation of mRNA splicing via spliceosome. Heatmaps showing differential changes of transcripts, proteins and phosphorylation sites for **C.** Hippo signaling **D.** Regulation of Hippo signaling, **E.** DNA damage response, **F.** DNA repair, **G.** Protein ubiquitination, **H.** Regulation of protein mono and polyubiquitination **I.** Graph showing protein expression levels of Ubiquitin (Ub) E2 and E3 ligases in response to SARS-CoV-2 infection. **J.** Heatmap showing changes in proteins involved in regulation of the cell cycle.

Supplementary Figure S7 related to Figure 6. Heatmaps showing **A.** transcriptome, proteome **B.** phosphoproteome, and **C.** acetylome changes in genes/proteins belonging to glycolysis, TCA cycle and amino acid metabolism pathways in response to SARS-CoV-2 infection.

Gassen, N.C., Papies, J., Bajaj, T., Emanuel, J., Dethloff, F., Chua, R.L., Trimpert, J., Heinemann, N., Niemeyer, C., Weege, F., et al. (2021). SARS-CoV-2-mediated dysregulation of metabolism and autophagy uncovers host-targeting antivirals. Nat Commun *12*, 3818. 10.1038/s41467-021-24007-w.

Zhang, Y., Guo, R., Kim, S.H., Shah, H., Zhang, S., Liang, J.H., Fang, Y., Gentili, M., Leary, C.N.O., Elledge, S.J., et al. (2021). SARS-CoV-2 hijacks folate and one-carbon metabolism for viral replication. Nat Commun *12*, 1676. 10.1038/s41467-021-21903-z.

Figure S1

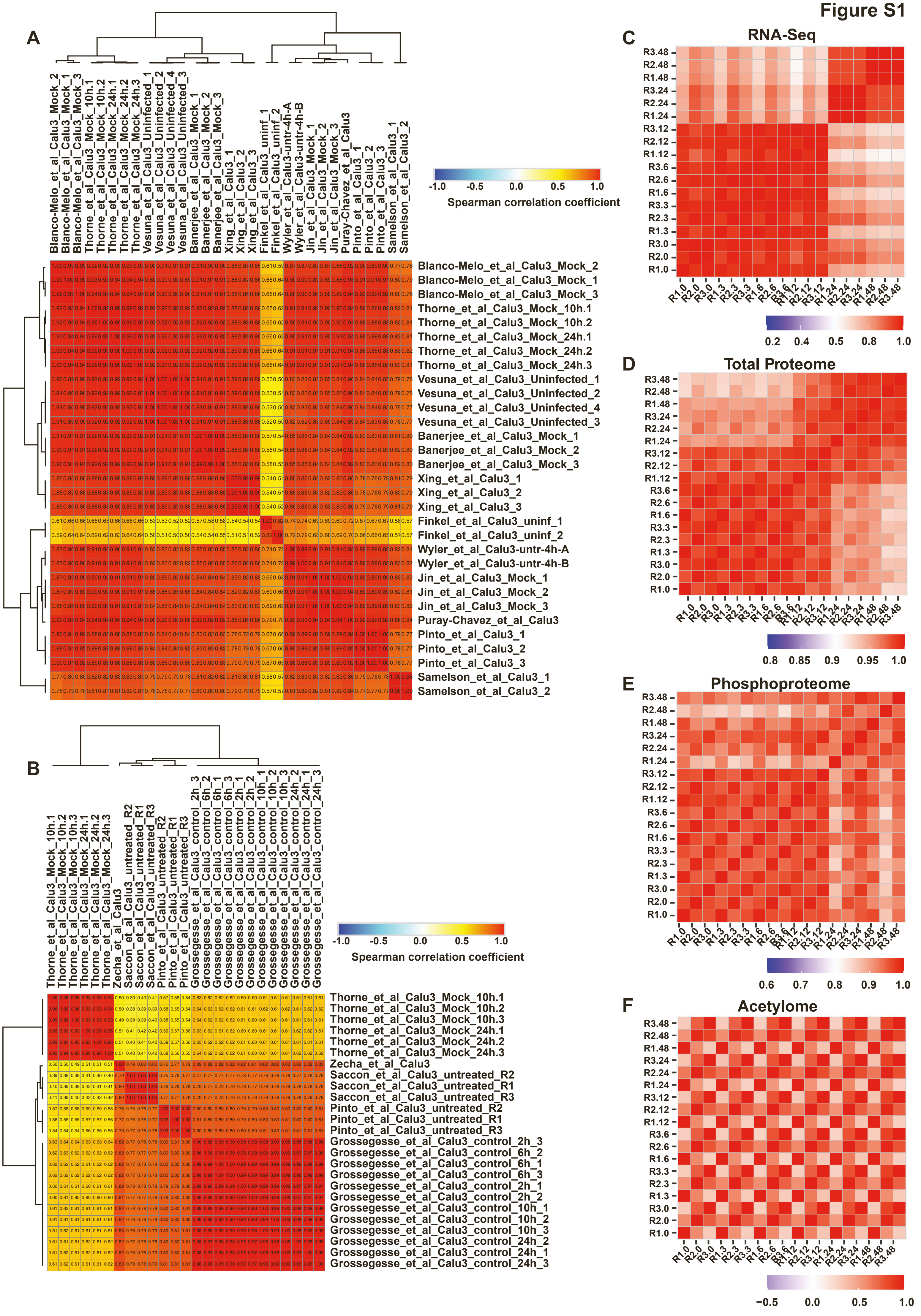
A
B
C
D
E
F
Spearman correlation coefficient
-1.0 -0.5 0.0 0.5 1.0
Blanco-Melo_et_al_Calu3_Mock_2
Blanco-Melo_et_al_Calu3_Mock_1
Blanco-Melo_et_al_Calu3_Mock_3
Thorne_et_al_Calu3_Mock_10h.1
Thorne_et_al_Calu3_Mock_10h.2
Thorne_et_al_Calu3_Mock_24h.1
Thorne_et_al_Calu3_Mock_24h.2
Thorne_et_al_Calu3_Mock_24h.3
Vesuna_et_al_Calu3_Uninfected_1
Vesuna_et_al_Calu3_Uninfected_2
Vesuna_et_al_Calu3_Uninfected_4
Vesuna_et_al_Calu3_Uninfected_3
Banerjee_et_al_Calu3_Mock_1
Banerjee_et_al_Calu3_Mock_2
Banerjee_et_al_Calu3_Mock_3
Xing_et_al_Calu3_1
Xing_et_al_Calu3_2
Xing_et_al_Calu3_3
Finkel_et_al_Calu3_uninf_1
Finkel_et_al_Calu3_uninf_2
Wyler_et_al_Calu3-untr-4h-A
Wyler_et_al_Calu3-untr-4h-B
Jin_et_al_Calu3_Mock_1
Jin_et_al_Calu3_Mock_2
Jin_et_al_Calu3_Mock_3
Puray-Chavez_et_al_Calu3
Pinto_et_al_Calu3_1
Pinto_et_al_Calu3_2
Pinto_et_al_Calu3_3
Samelson_et_al_Calu3_1
Samelson_et_al_Calu3_2
Thorne_et_al_Calu3_Mock_10h.1
Thorne_et_al_Calu3_Mock_10h.2
Thorne_et_al_Calu3_Mock_10h.3
Thorne_et_al_Calu3_Mock_24h.1
Thorne_et_al_Calu3_Mock_24h.2
Thorne_et_al_Calu3_Mock_24h.3
Zecha_et_al_Calu3
Saccon_et_al_Calu3_untreated_R2
Saccon_et_al_Calu3_untreated_R1
Saccon_et_al_Calu3_untreated_R3
Pinto_et_al_Calu3_untreated_R2
Pinto_et_al_Calu3_untreated_R1
Pinto_et_al_Calu3_untreated_R3
Grossegesse_et_al_Calu3_control_2h_3
Grossegesse_et_al_Calu3_control_6h_2
Grossegesse_et_al_Calu3_control_6h_1
Grossegesse_et_al_Calu3_control_6h_3
Grossegesse_et_al_Calu3_control_2h_1
Grossegesse_et_al_Calu3_control_2h_2
Grossegesse_et_al_Calu3_control_10h_1
Grossegesse_et_al_Calu3_control_10h_2
Grossegesse_et_al_Calu3_control_10h_3
Grossegesse_et_al_Calu3_control_24h_2
Grossegesse_et_al_Calu3_control_24h_1
Grossegesse_et_al_Calu3_control_24h_3
RNA-Seq
0.2 0.4 0.5 0.8 1.0
Total Proteome
0.8 0.85 0.9 0.95 1.0
Phosphoproteome
0.6 0.7 0.8 0.9 1.0
Acetylome
-0.5 0.0 0.5 1.0
R3.48
R2.48
R1.48
R3.24
R2.24
R1.24
R3.12
R2.12
R1.12
R3.6
R2.6
R1.6
R3.3
R2.3
R1.3
R3.0
R2.0
R1.0

Figure S2

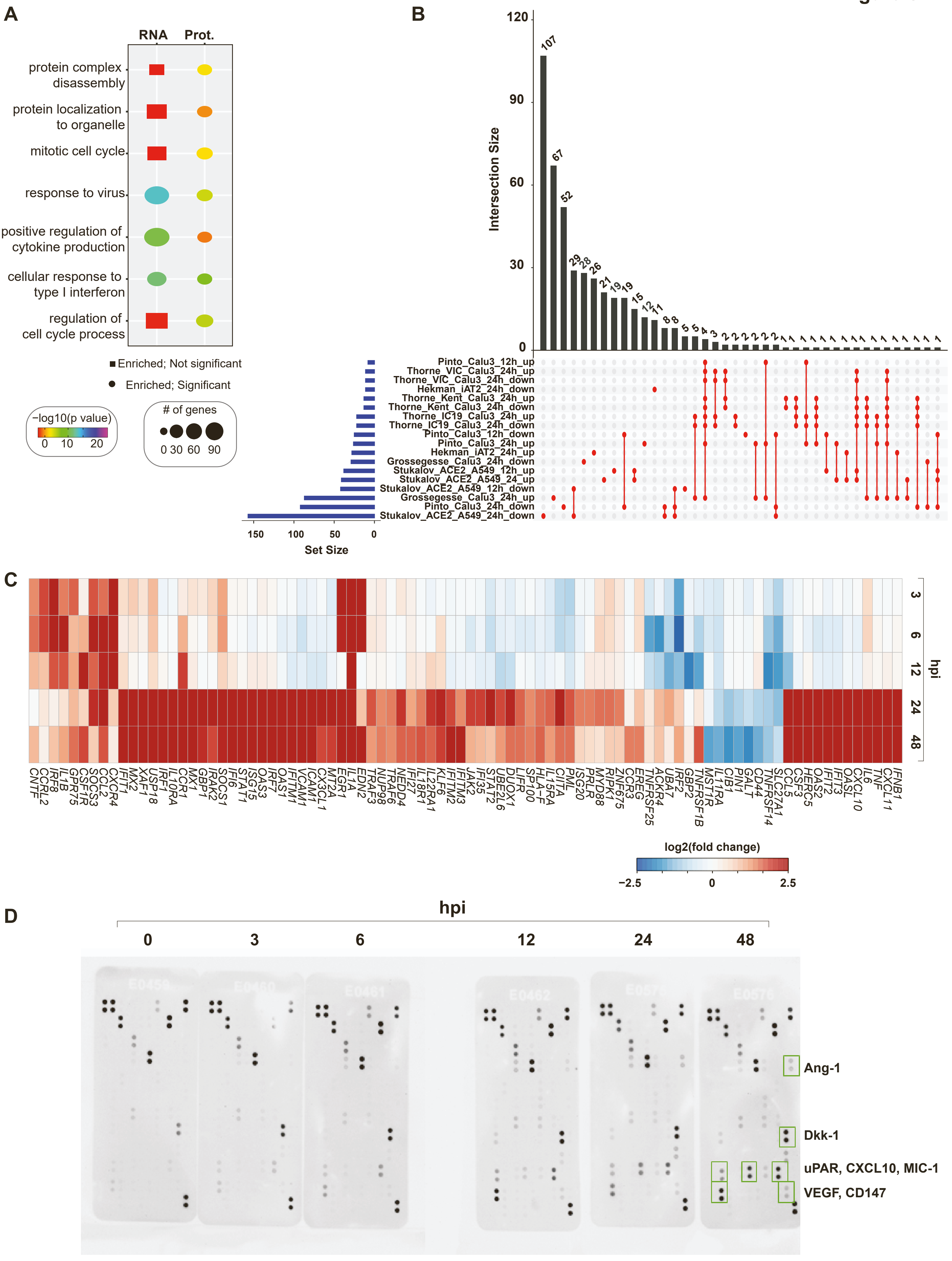
A
RNA
Prot.
protein complex disassembly
protein localization to organelle
mitotic cell cycle
response to virus
positive regulation of cytokine production
cellular response to type I interferon
regulation of cell cycle process
Enriched; Not significant
Enriched; Significant
−log10(p value)
# of genes
B
Intersection Size
Set Size
Pinto_Calu3_12h_up
Thorne_VIC_Calu3_24h_up
Thorne_VIC_Calu3_24h_down
Hekman_iAT2_24h_down
Thorne_Kent_Calu3_24h_up
Thorne_Kent_Calu3_24h_down
Thorne_IC19_Calu3_24h_up
Thorne_IC19_Calu3_24h_down
Pinto_Calu3_12h_down
Pinto_Calu3_24h_up
Hekman_iAT2_24h_up
Grossegesse_Calu3_24h_down
Stukalov_ACE2_A549_12h_up
Stukalov_ACE2_A549_24_up
Stukalov_ACE2_A549_12h_down
Grossegesse_Calu3_24h_up
Pinto_Calu3_24h_down
Stukalov_ACE2_A549_24h_down
C
hpi
log2(fold change)
D
hpi
0 3 6 12 24 48
Ang-1
Dkk-1
uPAR, CXCL10, MIC-1
VEGF, CD147

Figure S3

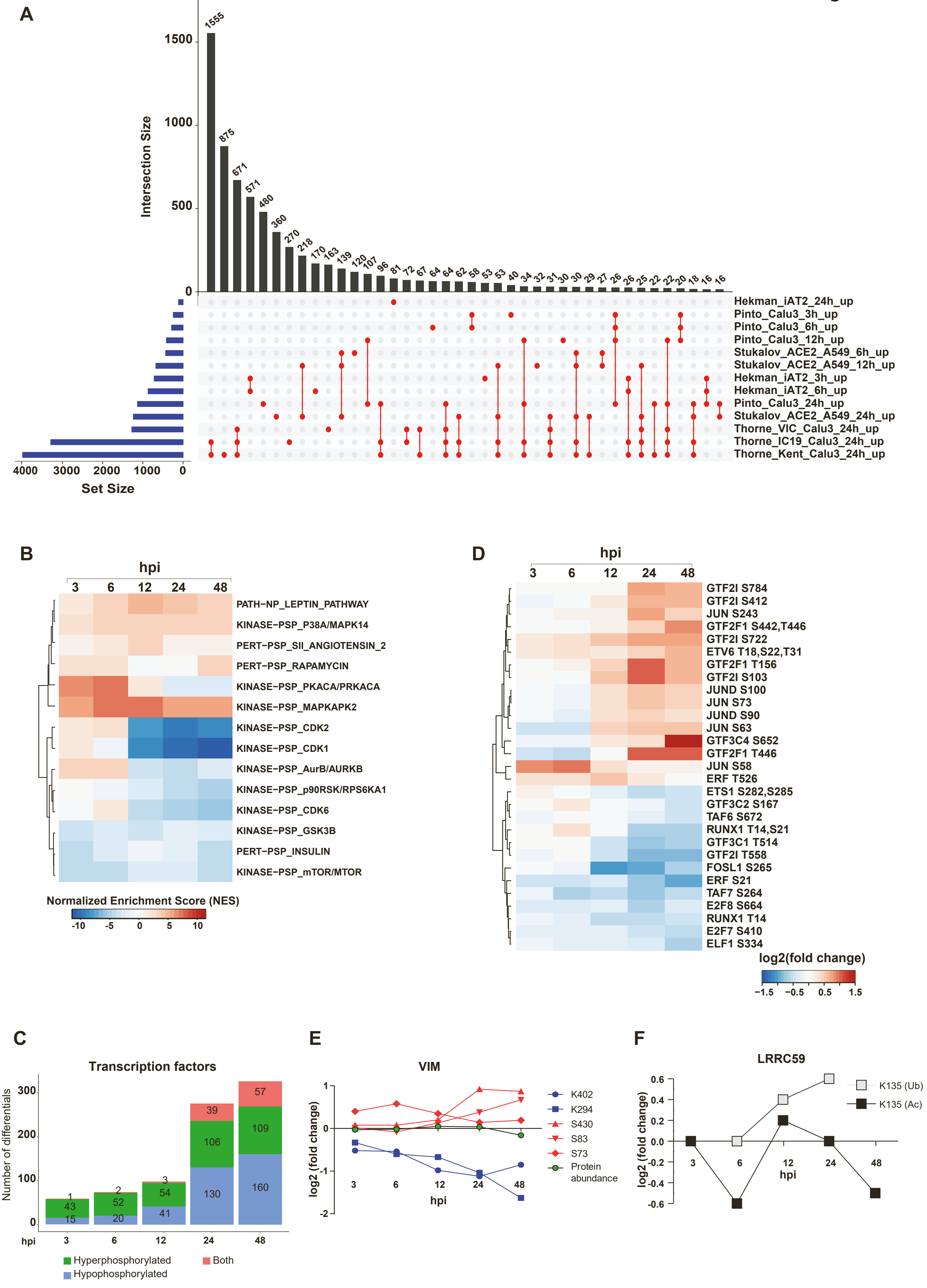
A
Intersection Size
1555
875
671
571
480
360
270
218
170
163
139
120
107
96
81
72
67
64
64
62
58
53
53
40
34
32
31
30
30
29
27
26
26
25
22
22
20
18
16
16
Hekman_iAT2_24h_up
Pinto_Calu3_3h_up
Pinto_Calu3_6h_up
Pinto_Calu3_12h_up
Stukalov_ACE2_A549_6h_up
Stukalov_ACE2_A549_12h_up
Hekman_iAT2_3h_up
Hekman_iAT2_6h_up
Pinto_Calu3_24h_up
Stukalov_ACE2_A549_24h_up
Thorne_VIC_Calu3_24h_up
Thorne_IC19_Calu3_24h_up
Thorne_Kent_Calu3_24h_up
Set Size
B
hpi
3 6 12 24 48
PATH−NP_LEPTIN_PATHWAY
KINASE−PSP_P38A/MAPK14
PERT−PSP_SII_ANGIOTENSIN_2
PERT−PSP_RAPAMYCIN
KINASE−PSP_PKACA/PRKACA
KINASE−PSP_MAPKAPK2
KINASE−PSP_CDK2
KINASE−PSP_CDK1
KINASE−PSP_AurB/AURKB
KINASE−PSP_p90RSK/RPS6KA1
KINASE−PSP_CDK6
KINASE−PSP_GSK3B
PERT−PSP_INSULIN
KINASE−PSP_mTOR/MTOR
Normalized Enrichment Score (NES)
-10 -5 0 5 10
D
hpi
3 6 12 24 48
GTF2I S784
GTF2I S412
JUN S243
GTF2F1 S442,T446
GTF2I S722
ETV6 T18,S22,T31
GTF2F1 T156
GTF2I S103
JUND S100
JUN S73
JUND S90
JUN S63
GTF3C4 S652
GTF2F1 T446
JUN S58
ERF T526
ETS1 S282,S285
GTF3C2 S167
TAF6 S672
RUNX1 T14,S21
GTF3C1 T514
GTF2I T558
FOSL1 S265
ERF S21
TAF7 S264
E2F8 S664
RUNX1 T14
E2F7 S410
ELF1 S334
log2(fold change)
−1.5 −0.5 0.5 1.5
C
Transcription factors
Number of differentials
hpi
3 6 12 24 48
1 43 15
2 52 20
3 54 41
39 106 130
57 109 160
Hyperphosphorylated
Hypophosphorylated
Both
E
VIM
log2 (fold change)
hpi
K402
K294
S430
S83
S73
Protein abundance
F
LRRC59
log2 (fold change)
hpi
K135 (Ub)
K135 (Ac)

Figure S4

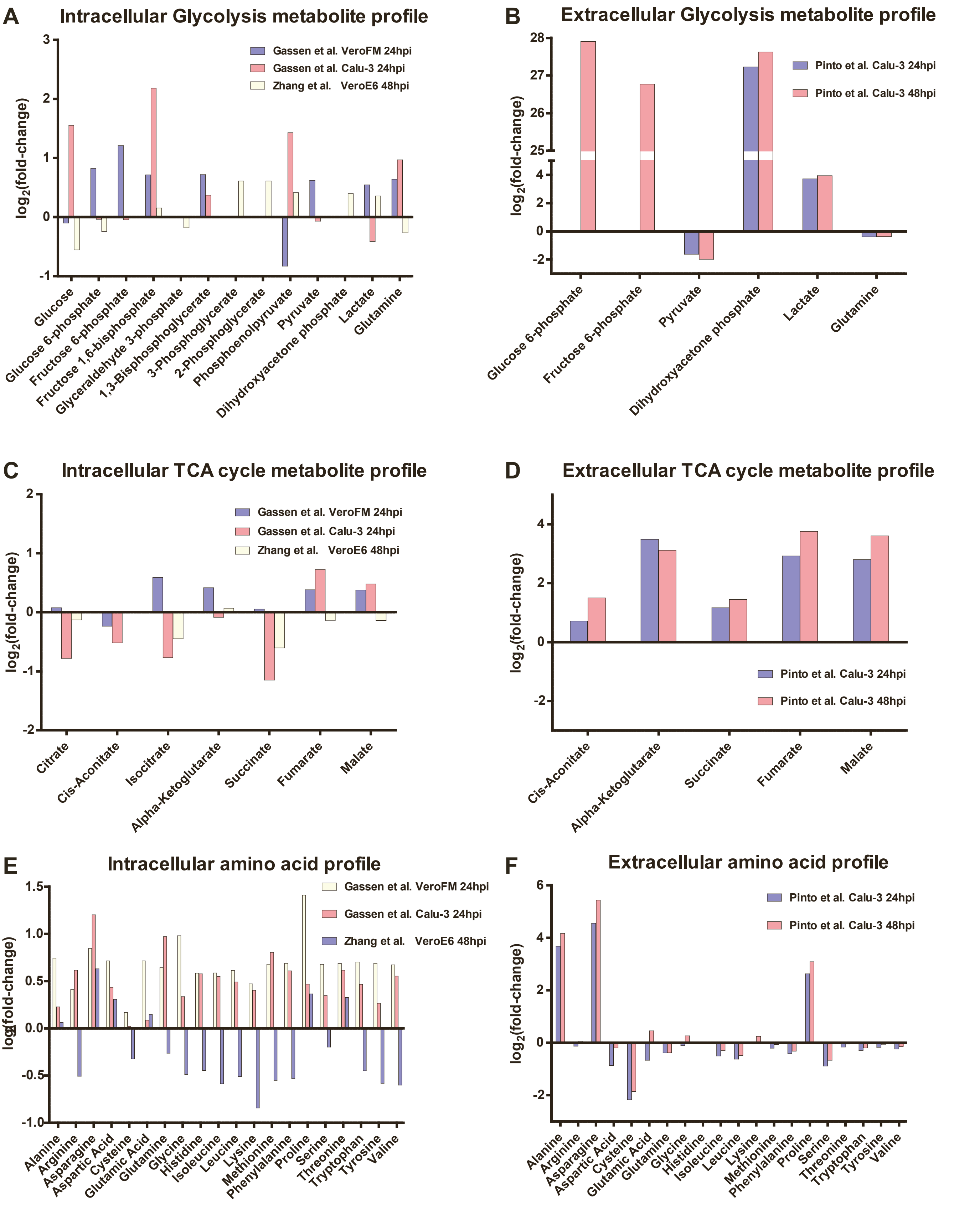
A
Intracellular Glycolysis metabolite profile
Gassen et al. VeroFM 24hpi
Gassen et al. Calu-3 24hpi
Zhang et al. VeroE6 48hpi
log2(fold-change)
3
2
1
0
-1
Glucose
Glucose 6-phosphate
Fructose 6-phosphate
Fructose 1,6-bisphosphate
Glyceraldehyde 3-phosphate
1,3-Bisphosphoglycerate
3-Phosphoglycerate
2-Phosphoglycerate
Phosphoenolpyruvate
Pyruvate
Dihydroxyacetone phosphate
Lactate
Glutamine
B
Extracellular Glycolysis metabolite profile
Pinto et al. Calu-3 24hpi
Pinto et al. Calu-3 48hpi
log2(fold-change)
28
27
26
25
4
2
0
-2
Glucose 6-phosphate
Fructose 6-phosphate
Pyruvate
Dihydroxyacetone phosphate
Lactate
Glutamine
C
Intracellular TCA cycle metabolite profile
Gassen et al. VeroFM 24hpi
Gassen et al. Calu-3 24hpi
Zhang et al. VeroE6 48hpi
log2(fold-change)
2
1
0
-1
-2
Citrate
Cis-Aconitate
Isocitrate
Alpha-Ketoglutarate
Succinate
Fumarate
Malate
D
Extracellular TCA cycle metabolite profile
Pinto et al. Calu-3 24hpi
Pinto et al. Calu-3 48hpi
log2(fold-change)
4
2
0
-2
Cis-Aconitate
Alpha-Ketoglutarate
Succinate
Fumarate
Malate
E
Intracellular amino acid profile
Gassen et al. VeroFM 24hpi
Gassen et al. Calu-3 24hpi
Zhang et al. VeroE6 48hpi
log2(fold-change)
1.5
1.0
0.5
0.0
-0.5
-1.0
Alanine
Arginine
Asparagine
Aspartic Acid
Cysteine
Glutamic Acid
Glutamine
Glycine
Histidine
Isoleucine
Leucine
Lysine
Methionine
Phenylalanine
Proline
Serine
Threonine
Tryptophan
Tyrosine
Valine
F
Extracellular amino acid profile
Pinto et al. Calu-3 24hpi
Pinto et al. Calu-3 48hpi
log2(fold-change)
6
4
2
0
-2
Alanine
Arginine
Asparagine
Aspartic Acid
Cysteine
Glutamic Acid
Glutamine
Glycine
Histidine
Isoleucine
Leucine
Lysine
Methionine
Phenylalanine
Proline
Serine
Threonine
Tryptophan
Tyrosine
Valine

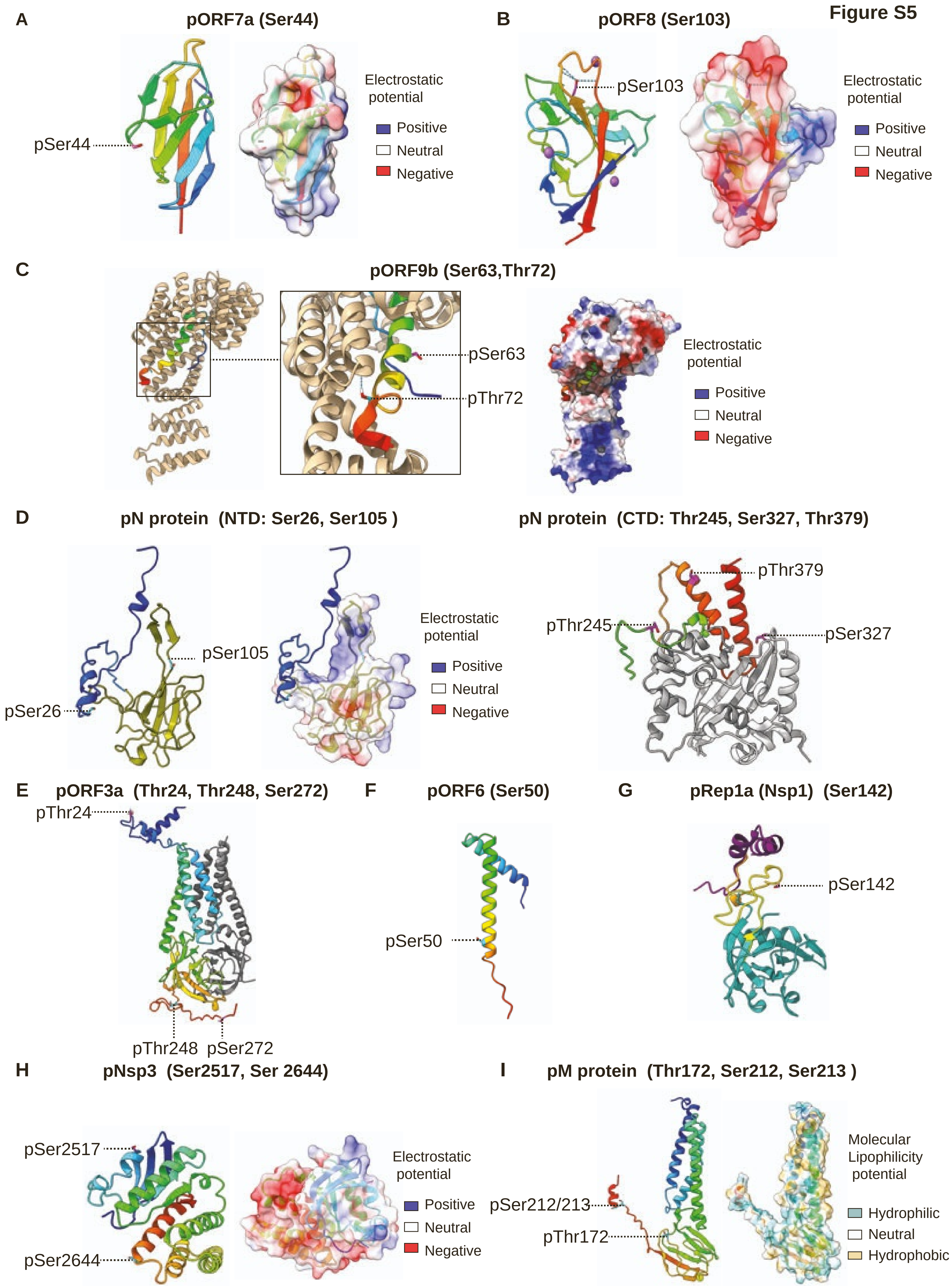
Figure S5
A
pORF7a (Ser44)
pSer44
Electrostatic potential
Positive
Neutral
Negative
B
pORF8 (Ser103)
pSer103
Electrostatic potential
Positive
Neutral
Negative
C
pORF9b (Ser63,Thr72)
pSer63
pThr72
Electrostatic potential
Positive
Neutral
Negative
D
pN protein (NTD: Ser26, Ser105)
pSer105
pSer26
Electrostatic potential
Positive
Neutral
Negative
pN protein (CTD: Thr245, Ser327, Thr379)
pThr379
pThr245
pSer327
E
pORF3a (Thr24, Thr248, Ser272)
pThr24
pThr248
pSer272
F
pORF6 (Ser50)
pSer50
G
pRep1a (Nsp1) (Ser142)
pSer142
H
pNsp3 (Ser2517, Ser 2644)
pSer2517
pSer2644
Electrostatic potential
Positive
Neutral
Negative
I
pM protein (Thr172, Ser212, Ser213)
pSer212/213
pThr172
Molecular Lipophilicity potential
Hydrophilic
Neutral
Hydrophobic

Figure S6

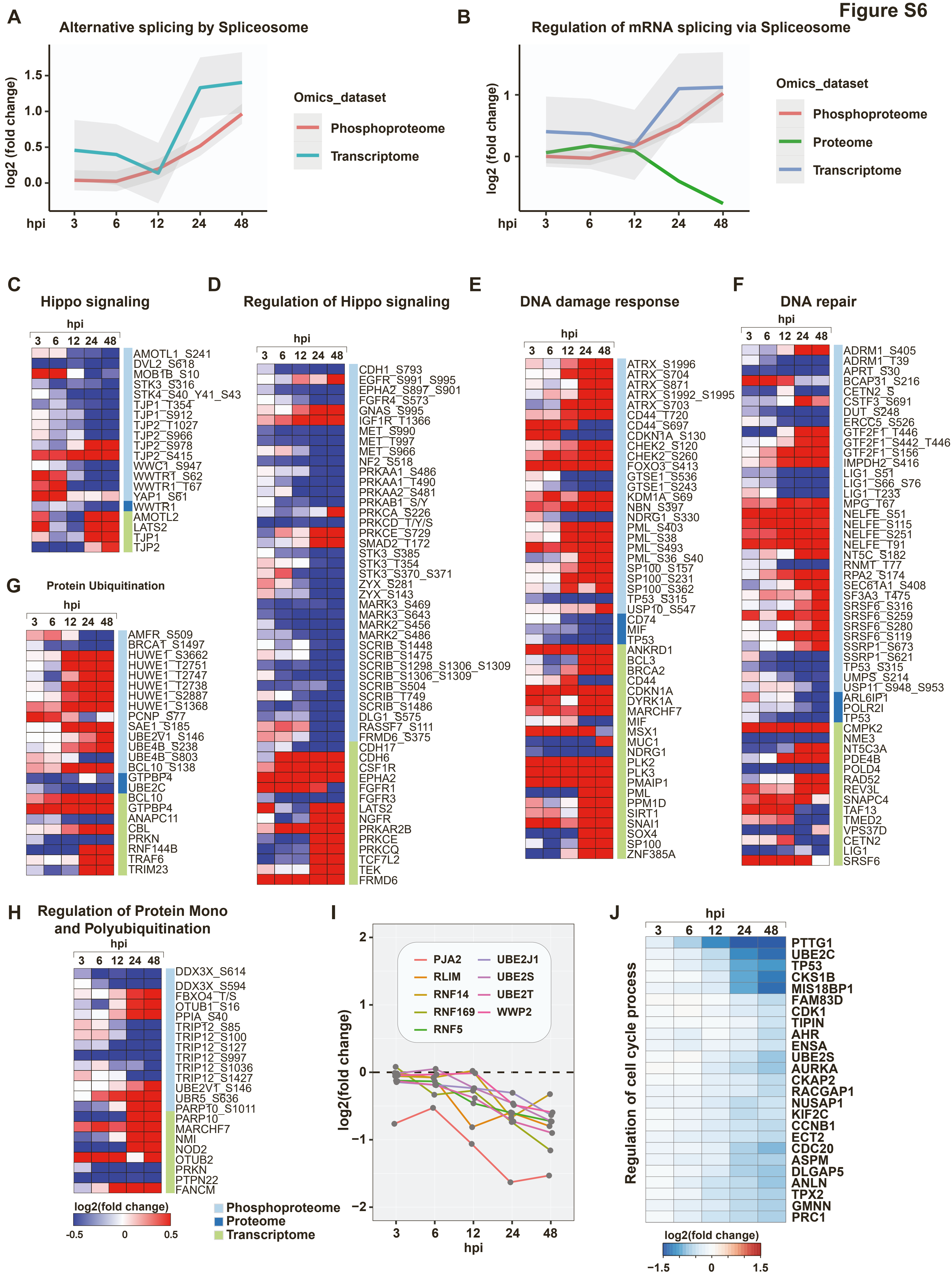
A
Alternative splicing by Spliceosome
log2 (fold change)
1.5
1.0
0.5
0.0
hpi 3 6 12 24 48
Omics_dataset
Phosphoproteome
Transcriptome
B
Regulation of mRNA splicing via Spliceosome
log2 (fold change)
1
0
hpi 3 6 12 24 48
Omics_dataset
Phosphoproteome
Proteome
Transcriptome
C
Hippo signaling
hpi
3 6 12 24 48
AMOTL1_S241
DVL2_S618
MOB1B_S10
STK3_S316
STK4_S40_Y41_S43
TJP1_T354
TJP1_S912
TJP2_T1027
TJP2_S966
TJP2_S978
TJP2_S415
WWC1_S947
WWTR1_S62
WWTR1_T67
YAP1_S61
WWTR1
AMOTL2
LATS2
TJP1
TJP2
D
Regulation of Hippo signaling
hpi
3 6 12 24 48
CDH1_S793
EGFR_S991_S995
EPHA2_S897_S901
FGFR4_S573
GNAS_S995
IGF1R_T1366
MET_S990
MET_T997
MET_S966
NF2_S518
PRKAA1_S486
PRKAA1_T490
PRKAA2_S481
PRKAB1_S/Y
PRKCA_S226
PRKCD_T/Y/S
PRKCE_S729
SMAD2_T172
STK3_S385
STK3_T354
STK3_S370_S371
ZYX_S281
ZYX_S143
MARK3_S469
MARK3_S643
MARK2_S456
MARK2_S486
SCRIB_S1448
SCRIB_S1475
SCRIB_S1298_S1306_S1309
SCRIB_S1306_S1309
SCRIB_S504
SCRIB_T749
SCRIB_S1486
DLG1_S575
RASSF7_S111
FRMD6_S375
CDH17
CDH6
CSF1R
EPHA2
FGFR1
FGFR3
LATS2
NGFR
PRKAR2B
PRKCE
PRKCQ
TCF7L2
TEK
FRMD6
E
DNA damage response
hpi
3 6 12 24 48
ATRX_S1996
ATRX_S704
ATRX_S871
ATRX_S1992_S1995
ATRX_S703
CD44_T720
CD44_S697
CDKN1A_S130
CHEK2_S120
CHEK2_S260
FOXO3_S413
GTSE1_S536
GTSE1_S243
KDM1A_S69
NBN_S397
NDRG1_S330
PML_S403
PML_S38
PML_S493
PML_S36_S40
SP100_S157
SP100_S231
SP100_S362
TP53_S315
USP10_S547
CD74
MIF
TP53
ANKRD1
BCL3
BRCA2
CD44
CDKN1A
DYRK1A
MARCHF7
MIF
MSX1
MUC1
NDRG1
PLK2
PLK3
PMAIP1
PML
PPM1D
SIRT1
SNAI1
SOX4
SP100
ZNF385A
F
DNA repair
hpi
3 6 12 24 48
ADRM1_S405
ADRM1_T39
APRT_S30
BCAP31_S216
CETN2_S
CSTF3_S691
DUT_S248
ERCC5_S526
GTF2F1_T446
GTF2F1_S442_T446
GTF2F1_S156
IMPDH2_S416
LIG1_S51
LIG1_S66_S76
LIG1_T233
MPG_T67
NELFE_S51
NELFE_S115
NELFE_S251
NELFE_T91
NT5C_S182
RNMT_T77
RPA2_S174
SEC61A1_S408
SF3A3_T475
SRSF6_S316
SRSF6_S259
SRSF6_S280
SRSF6_S119
SSRP1_S673
SSRP1_S621
TP53_S315
UMPS_S214
USP11_S948_S953
ARL6IP1
POLR2I
TP53
CMPK2
NME3
NT5C3A
PDE4B
POLD4
RAD52
REV3L
SNAPC4
TAF13
TMED2
VPS37D
CETN2
LIG1
SRSF6
G
Protein Ubiquitination
hpi
3 6 12 24 48
AMFR_S509
BRCA1_S1497
HUWE1_S3662
HUWE1_T2751
HUWE1_T2747
HUWE1_T2738
HUWE1_S2887
HUWE1_S1368
PCNP_S77
SAE1_S185
UBE2V1_S146
UBE4B_S238
UBE4B_S803
BCL10_S138
GTPBP4
UBE2C
BCL10
GTPBP4
ANAPC11
CBL
PRKN
RNF144B
TRAF6
TRIM23
H
Regulation of Protein Mono and Polyubiquitination
hpi
3 6 12 24 48
DDX3X_S614
DDX3X_S594
FBXO4_T/S
OTUB1_S16
PPIA_S40
TRIP12_S85
TRIP12_S100
TRIP12_S127
TRIP12_S997
TRIP12_S1036
TRIP12_S1427
UBE2V1_S146
UBR5_S636
PARP10_S1011
PARP10
MARCHF7
NMI
NOD2
OTUB2
PRKN
PTPN22
FANCM
log2(fold change)
-0.5 0 0.5
Phosphoproteome
Proteome
Transcriptome
I
2
1
0
−1
−2
log2(fold change)
PJA2
RLIM
RNF14
RNF169
RNF5
UBE2J1
UBE2S
UBE2T
WWP2
3 6 12 24 48
hpi
J
hpi
3 6 12 24 48
Regulation of cell cycle process
PTTG1
UBE2C
TP53
CKS1B
MIS18BP1
FAM83D
CDK1
TIPIN
AHR
ENSA
UBE2S
AURKA
CKAP2
RACGAP1
NUSAP1
KIF2C
CCNB1
ECT2
CDC20
ASPM
DLGAP5
ANLN
TPX2
GMNN
PRC1
log2(fold change)
−1.5 0 1.5

Figure S7

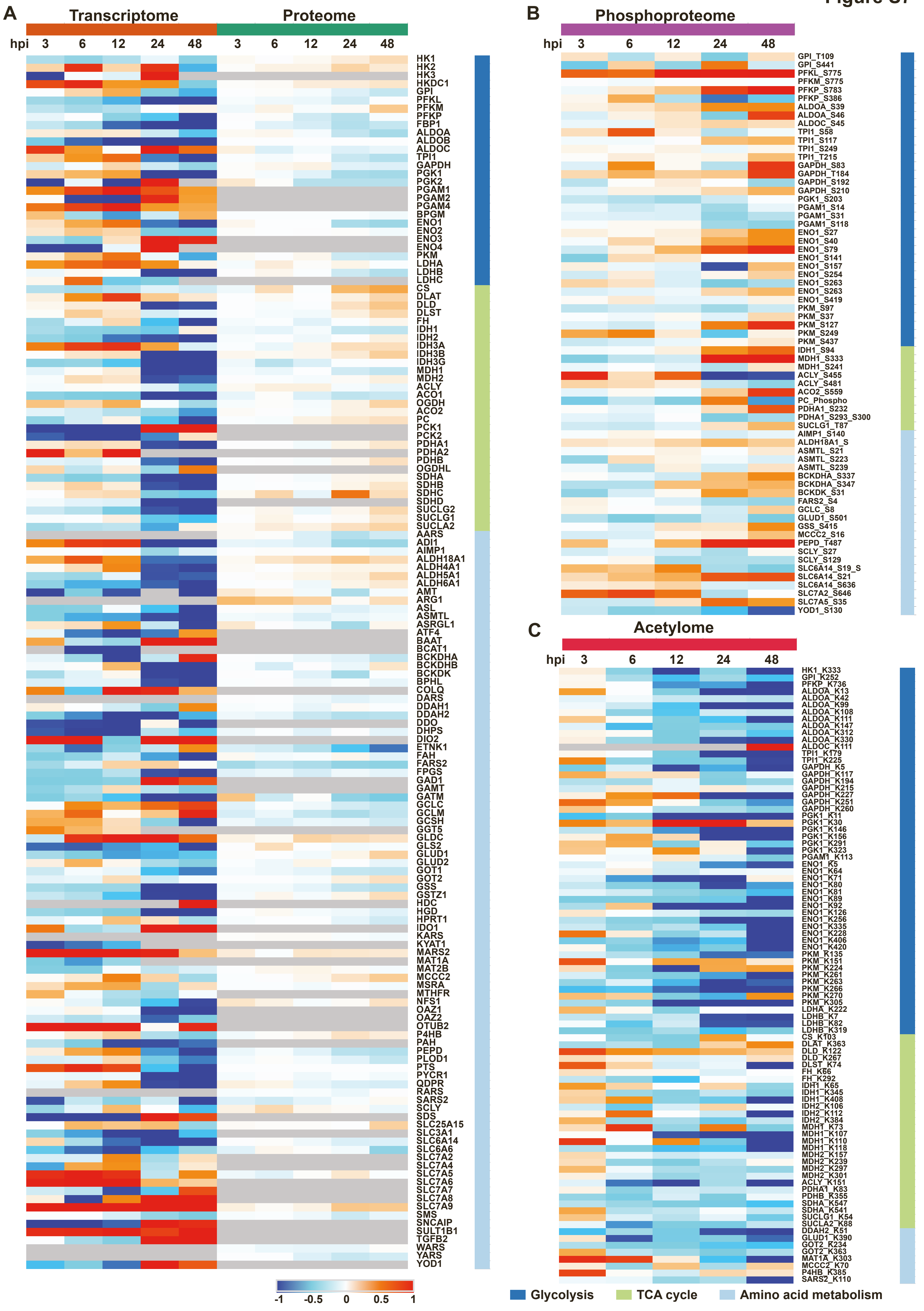
A
Transcriptome
Proteome
hpi 3 6 12 24 48 3 6 12 24 48
HK1 HK2 HK3 HKDC1 GPI PFKL PFKM PFKP FBP1 ALDOA ALDOB ALDOC TPI1 GAPDH PGK1 PGK2 PGAM1 PGAM2 PGAM4 BPGM ENO1 ENO2 ENO3 ENO4 PKM LDHA LDHB LDHC
CS DLAT DLD DLST FH IDH1 IDH2 IDH3A IDH3B IDH3G MDH1 MDH2 ACLY ACO1 OGDH ACO2 PC PCK1 PCK2 PDHA1 PDHA2 PDHB OGDHL SDHA SDHB SDHC SDHD SUCLG2 SUCLG1 SUCLA2
AARS ADI1 AIMP1 ALDH18A1 ALDH4A1 ALDH5A1 ALDH6A1 AMT ARG1 ASL ASMTL ASRGL1 ATF4 BAAT BCAT1 BCKDHA BCKDHB BCKDK BPHL COLQ DARS DDAH1 DDAH2 DDO DHPS DIO2 ETNK1 FAH FARS2 FPGS GAD1 GAMT GATM GCLC GCLM GCSH GGT5 GLDC GLS2 GLUD1 GLUD2 GOT1 GOT2 GSS GSTZ1 HDC HGD HPRT1 IDO1 KARS KYAT1 MARS2 MAT1A MAT2B MCCC2 MSRA MTHFR NFS1 OAZ1 OAZ2 OTUB2 P4HB PAH PEPD PLOD1 PTS PYCR1 QDPR RARS SARS2 SCLY SDS SLC25A15 SLC3A1 SLC6A14 SLC6A6 SLC7A2 SLC7A4 SLC7A5 SLC7A6 SLC7A7 SLC7A8 SLC7A9 SMS SNCAIP SULT1B1 TGFB2 WARS YARS YOD1
-1 -0.5 0 0.5 1
B
Phosphoproteome
hpi 3 6 12 24 48
GPI_T109 GPI_S441 PFKL_S775 PFKM_S775 PFKP_S783 PFKP_S386 ALDOA_S39 ALDOA_S46 ALDOC_S45 TPI1_S58 TPI1_S117 TPI1_S249 TPI1_T215 GAPDH_S83 GAPDH_T184 GAPDH_S192 GAPDH_S210 PGK1_S203 PGAM1_S14 PGAM1_S31 PGAM1_S118 ENO1_S27 ENO1_S40 ENO1_S79 ENO1_S141 ENO1_S157 ENO1_S254 ENO1_S263 ENO1_S263 ENO1_S419 PKM_S97 PKM_S37 PKM_S127 PKM_S249 PKM_S437
IDH1_S94 MDH1_S333 MDH1_S241 ACLY_S455 ACLY_S481 ACO2_S559 PC_Phospho PDHA1_S232 PDHA1_S293_S300 SUCLG1_T87
AIMP1_S140 ALDH18A1_S ASMTL_S21 ASMTL_S223 ASMTL_S239 BCKDHA_S337 BCKDHA_S347 BCKDK_S31 FARS2_S4 GCLC_S8 GLUD1_S501 GSS_S415 MCCC2_S16 PEPD_T487 SCLY_S27 SCLY_S129 SLC6A14_S19_S SLC6A14_S21 SLC6A14_S636 SLC7A2_S646 SLC7A5_S35 YOD1_S130
C
Acetylome
hpi 3 6 12 24 48
HK1_K333 GPI_K252 PFKP_K736 ALDOA_K13 ALDOA_K42 ALDOA_K99 ALDOA_K108 ALDOA_K111 ALDOA_K147 ALDOA_K312 ALDOA_K330 ALDOC_K111 TPI1_K179 TPI1_K225 GAPDH_K5 GAPDH_K117 GAPDH_K194 GAPDH_K215 GAPDH_K227 GAPDH_K251 GAPDH_K260 PGK1_K11 PGK1_K30 PGK1_K146 PGK1_K156 PGK1_K291 PGK1_K323 PGAM1_K113 ENO1_K5 ENO1_K64 ENO1_K71 ENO1_K80 ENO1_K81 ENO1_K89 ENO1_K92 ENO1_K126 ENO1_K256 ENO1_K335 ENO1_K228 ENO1_K406 ENO1_K420 PKM_K135 PKM_K151 PKM_K224 PKM_K261 PKM_K263 PKM_K266 PKM_K270 PKM_K305 LDHA_K222 LDHB_K7 LDHB_K82 LDHB_K319
CS_K103 DLAT_K363 DLD_K122 DLD_K267 DLST_K74 FH_K66 FH_K292 IDH1_K65 IDH1_K345 IDH1_K408 IDH2_K106 IDH2_K112 IDH2_K384 MDH1_K73 MDH1_K107 MDH1_K110 MDH1_K118 MDH2_K157 MDH2_K239 MDH2_K297 MDH2_K301 ACLY_K151 PDHA1_K83 PDHB_K355 SDHA_K547 SDHA_K541 SUCLG1_K54 SUCLA2_K88
DDAH2_K51 GLUD1_K390 GOT2_K234 GOT2_K363 MAT1A_K303 MCCC2_K70 P4HB_K385 SARS2_K110
Glycolysis
TCA cycle
Amino acid metabolism
